# Salvage LATTICE radiotherapy for a growing tumour despite conventional radio chemotherapy treatment of lung cancer

**DOI:** 10.1016/j.ctro.2022.11.016

**Published:** 2022-12-05

**Authors:** Rémy Kinj, Alessio Casutt, Tu Nguyen-Ngoc, Ange Mampuya, Luis Schiappacasse, Jean Bourhis, Constance Huck, David Patin, Maud Marguet, Michele Zeverino, Raphaël Moeckli, Michel Gonzalez, Alban Lovis, Mahmut Ozsahin

**Affiliations:** a Department of Radiation Oncology, Lausanne University Hospital (CHUV) and University of Lausanne (UNIL), Lausanne, Switzerland; b Department of Pulmonology, Lausanne University Hospital (CHUV) and Lausanne University (UNIL), Lausanne, Switzerland; c Department of Medical Oncology, Lausanne University Hospital (CHUV) and University of Lausanne (UNIL), Lausanne, Switzerland; d Institute of Radiation Physics, Lausanne University Hospital (CHUV) and University of Lausanne (UNIL), Lausanne, Switzerland; e Department of Thoracic Surgery, University Hospital Center of Lausanne (CHUV), and University of Lausanne (UNIL), Lausanne, Switzerland

**Keywords:** Lattice radiotherapy, Spatially fractionated radiotherapy, Immunotherapy, Lung cancer, Lattice boost, RT, Radiotherapy, IGRT, image-guided RT, SFRT, spatially fractionnated RT, LRT, lattice RT, NSCLC, non small-cell lung cancer

## Abstract

A 40-year-old patient with cT4cN1M0 squamous cell lung cancer of the upper right lobe received preoperative induction chemotherapy. Systemic induction treatment failed to reverse tumour growth with the addition of conventional radiotherapy (RT). A salvage lattice RT boost of 12 Gy was administered immediately to increase the dose to the tumour. Conventional RT was resumed at the planned dose of 60 Gy. The tumour shrank rapidly, and the patient was surged. The postoperative pathology remained ypT0ypN0 status.

## Highlights

• LATTICE radiotherapy (LRT) is a form of spatially fractionated radiotherapy inspired from GRID radiotherapy.
• LRT is usually used as a boost or in a palliative intent.
• LRT permits a dose escalation into the tumor while preserving surrounding organs at risk.
• LRT is presented in this report as a salvage boost treatment.

## 1 Introduction

Unresectable locally advanced lung cancer is treated with radiotherapy (RT) and chemotherapy, followed by immunotherapy [1], [2]. Implementation of image-guided RT (IGRT) enables target volume evolution during treatment. Adaptive RT (ART) and replanning may be considered if significant target volume modification and anatomical or physiological deviations from the initial simulation occur [3]. Fortunately, tumour volume progression is rare and has a poor prognosis for patients undergoing radiochemotherapy (RCT). Spatially fractionated RT (SFRT) in 2D GRID RT configuration was initially tested for palliative treatment of bulky tumours [4]. In 2010, SFRT was extended to a 3D configuration named lattice RT (LRT). LRT is based on the SFRT principle of focusing on high-dose regions called vertices. LRT can be delivered as a palliative or boost treatment. In the case of an LRT boost, the aim is to escalate the dose inside the tumour while preserving the surrounding organs at risk (OARs) through spatial fractionations and inward margins [5]. This case report describes the use of lattice salvage boost in a progressive young patient during lung RCT.

## 2 Case report

A 40-year-old male smoker with no medical history, PS 0, presented with a chronic dry cough that had evolved from October 2021. The patient presented with chronic stage II NYHA dyspnoea with new right parasternal pain radiating to the cervical and cerebral levels. At the systemic level, he reported grade 1 anorexia with a weight loss of 10 kg over the previous four months. A chest computed tomography (CT) scan was performed at the end of February 2022, revealing a voluminous lung mass of 10 cm centred on the upper right lobe, infiltrating the middle lobe scissors and the upper segment of the lower right lobe. It also revealed stenosis of the pulmonary arteries and veins with complete obstruction of the right upper lobar bronchus and sub-stenosis of the distal section of the main right bronchus.

The patient underwent a transthoracic biopsy, and histopathological results suggested lung adenocarcinoma, with TTF-1 and CK7 positive and p40 and CK20 negative. Positron emission tomography/computed tomography (PET/CT) revealed homolateral mediastinal nodal hypermetabolism without distant metastasis. The brain MRI was negative.

**Fig. 1:**
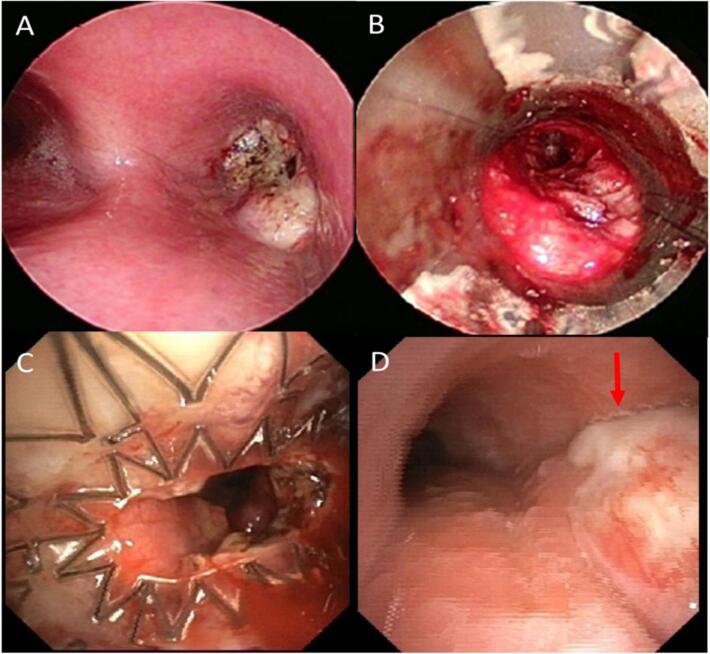
Images of the endobronchial assessments. A. Endobronchial main right bronchus complete obstruction by an endoluminal mass. B. Rigid bronchoscopy view of the right main bronchus during mechanical debulking. C. Main right bronchus permeability restored after Aero® stent placement. D. Tumour endobronchial progression occluding the stent and englobing the carina (red arrow). (For interpretation of the references to colour in this figure legend, the reader is referred to the web version of this article.)

In early March 2022, he underwent an endoscopic workup (rigid bronchoscopy and mediastinal endobronchial ultrasound with transbronchial needle aspiration [EBUS-TBNA]) for endobronchial extension and mediastinal assessment. Pulmonologists observed an endobronchial mass in the right main bronchus, reaching 1 cm from the main carina and completely blocked the right main bronchus (Fig. 1**A**). The patient underwent endobronchial debulking to restore the patency of the right bronchial tree (Fig. 1**B**). Biopsies of the endoluminal mass were repeated, histopathological analysis of the tumour mass showed a non-small-cell carcinoma (NSCLC), suggesting squamous-cell carcinoma (positive for p40 and CK-AE1/AE3 and negative for TTF1, Napsin A, SALL4 and CD34) with a pathological KRAS exon 2: mutation c.34G > T (p.Gly12Cys), in contrast to the results of the firsts samplings. EBUS-guided TBNAs at lymph node stations 4L, 4R, and 7R levels were lymph node representative and showed no tumour cells.

The cancer was re-classified as squamous cell lung cancer of the upper right lobe (cT4cN1cM0, stage IIIA). The thoracic oncological tumour board proposed neoadjuvant chemotherapy before surgery. The patient received initial treatment with carboplatin, paclitaxel, and pembrolizumab in mid-March 2022. After the initial treatment, at the end of March, the patient presented with increased dyspnoea in the context of a Covid-19 infection. A new CT scan revealed radiological deterioration with the appearance of important signs of subtotal bronchial occlusion of the right lung. New bronchoscopy showed an increase in the extrinsic compression component of the distal trachea, englobing the proximal section of the right main bronchus. An AERO stent (Merit Medical Systems, Utah, USA) was implanted (diameter, 10 mm; length, 40 mm) to re-establish the main right bronchus airway permeability after debulking (Fig. 1**C**). The addition of preoperative RT was decided due to tumour progression despite chemo-immunotherapy.

**Fig. 2:**
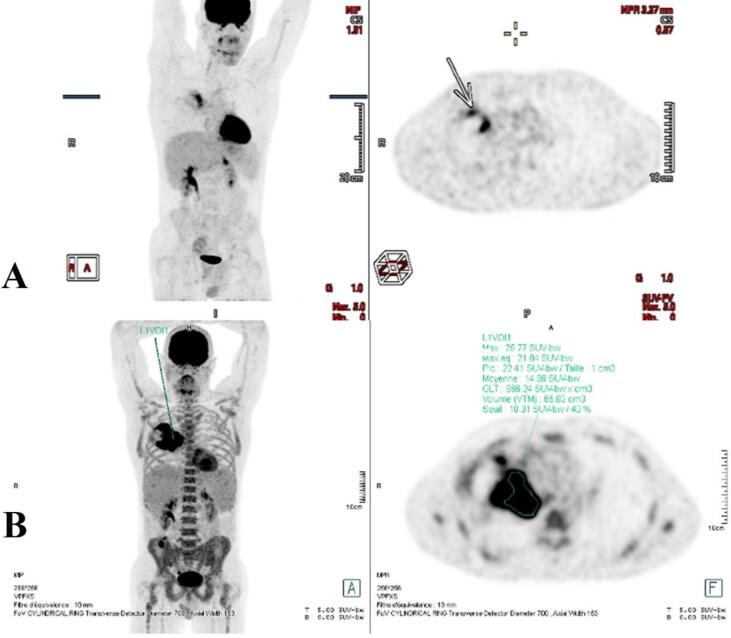
PET CT imaging A. at 1 month after the end of combined treatment and B. before combined treatment.

**Fig. 3:**
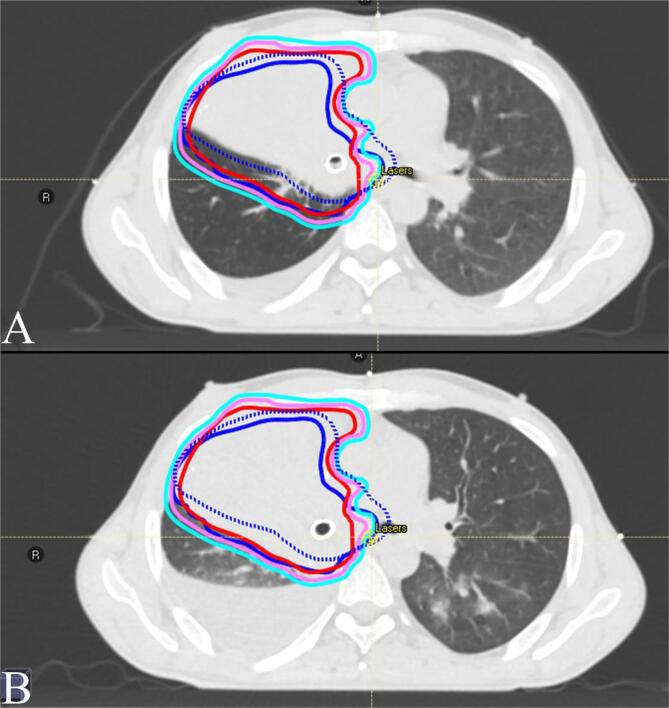
Planning CTs and illustrating tumour progression despite radio-chemo-immunotherapy. A. Initial planning CT (dark blue dashed line: initial planned PTV; dark blue solid lines: deformed PTV on initial CT; light blue: re-planned PTV on initial CT) B. Tumour progression, replanning CT after 10 Gy (light blue: replanned PTV; pink: replanned CTV; red: replanned GTV; dark blue dashed line: initial planned PTV; dark blue solid lines: deformed initial PTV). (For interpretation of the references to colour in this figure legend, the reader is referred to the web version of this article.)

**Fig. 4:**
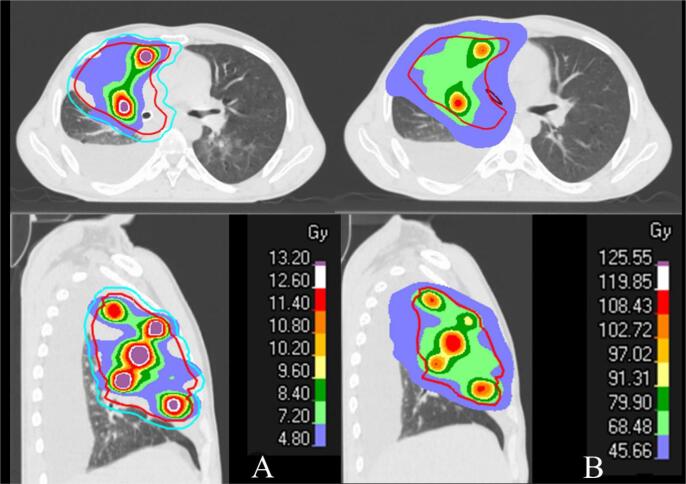
A. Salvage LRT boost, LRT plan B. EQD2 dose summation of the entire treatment.

At the end of April 2022, RT was started concomitantly with chemo-immunotherapy, targeting the right lung lesion. The plan was initially designed to deliver a dose of 60 Gy in 30 fractions **(**Fig. 2**A).** However, after 5 fractions of treatment, pre-treatment Cone-Beam CT imaging revealed tumour progression. On the same day, the patient underwent bronchoscopy to confirm tumour progression occluding the stent (Fig. 1**D**) and stenosis of the distal trachea up to 50 %. Laser-assisted de-obstruction was performed, enabling recovery of the stent. A new plan was urgently calculated and delivered after only 1 day off (Fig. 3). Because of this unexpected progression during radio-chemo-immunotherapy, we decided to urgently perform salvage dose escalation through an LRT single-fraction boost of 12 Gy. Medical oncologists also decided to switch from paclitaxel to concomitant vinorelbine chemotherapy. The LRT treatment was delivered the following Friday (to enhance recovery during the weekend) after 22 Gy of conventional RT. Seven vertices of 1.2–2.5 cm diameter were distributed into the GTV. The total volume of vertices represented 48 cc (3.8 %) encompassed in a PTV of 1252 cc. The prescription dose was isodose 80 %, corresponding to a Dmax of 15.3 Gy to the vertices, and the dose to the valleys was 2.0 Gy (Fig. 4**A**). An equivalent dose of 2 Gy EQD2 was used to validate the entire radiotherapy sequence. This spatial fractionation and heterogeneous distribution enabled the escalation of the dose to a Dmax of 116.4 Gy EQD2 in the tumour vertices, while preserving OARs. All standard dose constraints for the lungs, spinal canal, oesophagus, trachea, and main bronchus were met [6]. The maximal heart dose was slightly exceeded, with a Dmax of 63.7 Gy EQD2 (Fig. 4**B**). Following LRT, conventional RT was resumed, and the tumour rapidly responded to the LRT boost. The patient had to be re-planned one week after the LRT boost because of tumour regression, and a second revised plan could be resumed at 60 Gy. The bronchus stents could be removed a few days after the end of RT due to a good response and reopening of the bronchus airways.

A re-evaluation PET-CT in mid-July 2022, 1 month after the end of RT, showed a very good partial response **(**Fig. 2**B)**. The patient was presented to our oncological board, and thoracic surgeons proposed surgery. The patient underwent right pneumonectomy with mediastinal dissection. Post-operative histology revealed a pathological complete response of ypT0ypN0 (0/13). Post-operative course was uneventful, and the patient was discharged on post-operative day 8. Bronchial stumps did not present early or late insufficiency.

## 3 Discussion

This report describes the adaptive management of aggressive lung cancer in a young patient and the potential of salvage LRT boosts in reversing tumour progression. The LRT boost was designed to enable dose escalation within the GTV while limiting the dose to surrounding OARs. The use of EQD2 summation prevented dose constraints from being exceeded.

Since 2010, LRT has been reported in more than 150 patients in various locations [5]. LRT series in non-small cell lung cancer (NSCLC) are uncommon, and the dosimetric parameters appear comparable to our report **(**Table 1**)**. However, the novelty of this report is that LRT boost was used as salvage therapy in a patient with a growing tumour during treatment, despite concomitant chemo-immuno-radiotherapy. Salvage LRT boost was used in conjunction with chemotherapy, immunotherapy, and conventional radiotherapy, enabling a complete response as determined by ypT0ypN0 post-operative pathology. However, evaluating the extent to which the LRT salvage boost contributes to treatment success is challenging. Several biases have been identified. First, after the introduction of immunotherapy, it is common to observe pseudo-progression; an increase in tumour size can be observed due to immune infiltration into the tumour and cannot be distinguished from actual tumour growth [7]. Second, the systemic treatment was changed concurrently with the LRT boost implementation, so we could not evaluate whether the observed response was due to the switch of the systemic agent or the LRT salvage boost. Moreover, a response could have been observed later without an LRT boost due to the possibility of a delayed response, and the decision of an LRT salvage boost remains controversial.

**Table 1:** Largest series of LRT in non-small cell lung cancer.

Author/Year	Number of patients/Design	Aim/Sequence	Dose/Number of fractions	Average VerteicesVolume/GTV (%)	Average Vertices diameter (cm)/Average Number of vertices	Average Total dose in GTV/in Vertices	Average EQD2 dose in GTV/in Vertices
Amendola et al/2019 [15]	10/R	Curative/LRT before conventional RCT	18 Gy/1 fraction	1.3	1.0/4	72 Gy/87 Gy	76 Gy/117 Gy
Wu et al/2020 [5]	18/R	Palliative and curative	4.5–18 Gy/1–3	1.3	1.1/18	–	–
Larrea et al/2021 [17]	11/R	Curative/LRT before conventional RCT	15 Gy/1	–	1.0/6	–	–
Current Report	1/R	LRT boost during conventional RCT	12 Gy/1	3.8	1.3/7	67 Gy/74 Gy	74 Gy/103 Gy

However, combining LRT with a checkpoint inhibitor anti-PD1 drug could enhance the clinical response. SFRT with peak-valley and high-low dose alternation within the tumour volume may also be significant in mediating antitumour immune response. This dose alteration and heterogeneity could favour antitumour *T*-cell priming, and enhance radiation-induced immune modulation [8], [9], [10], [11]. Furthermore, low-dose areas in valleys may maintain the perfusion necessary for circulating factors contributing to antitumour immunity [12], [13], [14]. The interaction of LRT with the therapeutic effects of immune checkpoint inhibitors could be improved, leading to a complete pathological response. The release of more circulating antigens by a larger tumour volume may increase the probability of an abscopal effect [15], [16].

The dose of 12 Gy was calculated as a single fraction LRT boost while considering the dose constraints of OARs surrounding the tumour. We generally observe a quarter-to-half of the prescribed dose to the vertices distributed at the periphery of the PTV. In this case, 3–6 Gy in one fraction that corresponds to an EQD2 of 3.6–10.8 Gy, considering an α/β ratio of 3 Gy, and was expected to be tolerable for surrounding OARs. We calculated an EQD2 dose summation and all standard dose constraints for the lungs, spinal canal, oesophagus, trachea, and main bronchi, except for the maximal heart dose, which was slightly exceeded with a Dmax of 63.7 Gy EQD2. We did not expect late significant side effects since the dose constraints were followed using an EQD2 dose summation despite the short follow-up. A comparable range of LRT doses has been used in other studies treating NSCLC (Table 1).

Using salvage LRT boost appeared appropriate in this case and resulted in a favourable outcome. However, data on the use of LRT in clinical practice are limited due to lack of prospective trials, and the main reports are retrospective. In locally advanced NSCLC, it would be interesting to design a dose-escalation prospective trial comparing conventional RCT with conventional RCT associated with an LRT boost, followed by immunotherapy. The use of LRT boost in lung tumours should be reserved after case-by-case discussion.

## Declaration of Competing Interest

The authors declare that they have no known competing financial interests or personal relationships that could have appeared to influence the work reported in this paper.
